# Mushroom as a product and their role in mycoremediation

**DOI:** 10.1186/s13568-014-0029-8

**Published:** 2014-04-01

**Authors:** Shweta Kulshreshtha, Nupur Mathur, Pradeep Bhatnagar

**Affiliations:** 1 Amity Institute of Biotechnology, Amity University Rajasthan, 14- Gopal Bari, Ajmer Road, A-200, Vaishali Nagar, Jaipur 302021, Rajasthan, India; 2 Department of Zoology, University of Rajasthan, Jaipur 302005, JLN Marg, India; 3 Department of Life Sciences, The IIS University, Gurukul Marg, Jaipur 302020, Mansarovar, India

**Keywords:** Biodegradation, Bioremediation, Genotoxicity, Biosorption, Mushroom, Ames test, Product, Industrial waste, Agroindustrial waste, Bioconversion

## Abstract

Mushroom has been used for consumption as product for a long time due to their flavor and richness in protein. Mushrooms are also known as mycoremediation tool because of their use in remediation of different types of pollutants. Mycoremediation relies on the efficient enzymes, produced by mushroom, for the degradation of various types of substrate and pollutants. Besides waste degradation, mushroom produced a vendible product for consumption. However, sometimes they absorb the pollutant in their mycelium (biosorption process) and cannot be consumed due to absorbed toxicants. This article reviews the achievement and current status of mycoremediation technology based on mushroom cultivation for the remediation of waste and also emphasizes on the importance of mushroom as product. This critical review is also focused on the safety aspects of mushroom cultivation on waste.

## Introduction

Biological approaches based on industrial and environmental biotechnology is focusing on the development of “*clean technologies*" which emphasizes on the maximum production, reduced waste generation, treatment and conversion of waste in some useful form. Further, these clean technologies focus on the use of biological methods for the remediation of waste. One such biological method is *mycoremediation* which is based on the use of fungi and mushroom for the removal of waste from the environment. The mushrooms and other fungi possess *enzymatic machinery* for the degradation of waste/pollutants and therefore, can be applied for a wide variety of pollutants (Purnomo et al. [2013]; Kulshreshtha et al. [2013]). However, mushrooms, a basidiomycetous fungus, are becoming more popular nowadays for remediation purposes because it is not only a *bioremediation tool* but also provide mycelium or fruiting bodies as a *source of protein*. The efficiency of mushroom species in producing food protein in the form of biomass or fruiting bodies from different wastes lies in their ability to degrade waste via secretion of a variety of hydrolyzing and oxidizing enzymes (Kuforiji and Fasidi [2008]; Zhu et al. [2013]). This has attracted research attention in the field of mushroom cultivation and waste remediation.

Many reports have published to emphasize the role of mushroom in bioremediation of wastes by the process of *biodegradation, biosorption and bioconversion* (Akinyele et al. [2012], Kulshreshtha et al. [2013a]; Kumhomkul and Panich-pat [2013]; Lamrood and Ralegankar [2013]). Many scientists have studied the role of different enzymes in the degradation process; degradation products formed by it and conditions affecting the degradation process (Novotný et al. [2004]; Akinyele et al. [2011]; Zhu et al. [2013]). However, safety aspects of the process and products have not been reported so far. There is scarcity of reports indicating the pros and cons of mushroom cultivation on wastes and their further utilization as food. Moreover, *mushroom as a product* is meagerly reported.

Keeping this in mind, in this review we are discussing the use of *mushroom as a biological tool* for cleanup the environment. Mushroom is not only a mycoremediation tool but also a *product.* Mushroom fruiting bodies generated on industrial and agro-industrial wastes are considered as a product. We have also focused on the safety aspects of mushroom cultivation on waste.

### Mushroom as a product

Mushrooms are the product of biological origin and can be developed from biological wastes, agricultural wastes, agro-industrial wastes and industrial wastes. Besides this, these mushrooms can be used as a source of proteins, amino acids and several biological active molecules which not only provide nutrition but also use for therapeutic purposes (Table 1). Therefore, these can be considered as an important product.

**Table 1:** Role of mushroom as an important product

**S. no.**	**Mushroom**	**As a product**	**References**
1	*Pleurotus, Agaricus, Ganoderma Schizophyllan commune, Grifola frondosa Coriolus versicolor, Ganoderma lucidum,*	Used as medicine to boost immune responses against cancer	Kodama et al. ([2002]); Gao et al. ([2003]); Maehara et al. ([2012])
2	*Pleurotus, Agaricus,*	Possess antimutagenic or antigenotoxic power to fight against cancer	Gameiro et al. ([2013]); Kang et al. ([2012])
3	*Ganoderma lucidum*, *Phellinus rimosus*, *Pleurotus florida* and *Pleurotus pulmonaris*	Used as antioxidant and antitumor agent	Ajith and Janardhanan ([2007])
4	*Pleurotus, Agaricus*	Used as food	

Edible mushrooms are *highly nutritious* and can be compared with eggs, milk and meat (Oei [2003]). Mushroom is a protein rich food and has been considered as the source of single cell protein. These are easily digestible and possess a high amount of amino acids but lacks cholesterol. These possess high quantities of fibers, few sugars and low calories and a high quantity of the amino acids phenylalanine, threonine and tyrosine.

As far as the *nutrient profile* of mushroom are concerned, these are *influenced by many factors* including the type of substrate on which these are cultivated. There are some differences in the nutrient content of the mushroom cultivated on different substrates (Mabrouk and Ahwanyi [2008]; Akinyele et al. [2011]; Kulshreshtha et al. [2013b]). However, this change in nutritional content never found to affect their edibility. Therefore, it is still a beneficial technology because it solves two major problems simultaneously i.e. waste accumulation and shortage of proteinaceous food.

Besides, use for edible purpose, mushroom is used for other industrial processes like biopulping and biobleaching. Hence, the importance of this as product cannot be ignored.

### Mushroom as mycoremediation tool

Remediation through fungi is also called as mycoremediation. Mycoremediation tool refers to mushrooms and their enzymes due to having ability to degrade a wide variety of environmentally persistent pollutants, transform industrial and agro-industrial wastes into products.

### Mycoremediation potential of mushroom

Mushroom uses different methods to decontaminate polluted spots and stimulate the environment. These methods include - (i) Biodegradation (ii) Biosorption (iii) Bioconversion.

#### Biodegradation

The term ‘*Biodegradation*’ is used to describe the ultimate degradation and recycling of complex molecule to its mineral constituents. It is the process which leads to complete mineralization of the starting compound to simpler ones like CO_2_, H_2_O, NO_3_ and other inorganic compounds by living organisms. A lot of research has been done on the degradation abilities of mushroom and their enzymes and is depicted in Table 2. Many reports have been published on the compounds produced by degradation of various wastes and factor affecting the processes.

**Table 2:** Role of mushroom in degradation of pollutants

**S. no.**	**Mushroom spp.**	**Waste/Pollutants**	**Remarks**	**References**
1	*Pleurotus ostreatus*	Oxo-Biodegradable plastic	Mushrooms degraded the plastic and grew on it.	da Luz et al. ([2013])
2	*Lentinula edodes*	2,4-dichlorophenol	Mushrooms degraded 2,4-dichlorophenol (DCP) by using vanillin as an activator	Tsujiyama et al. ([2013])
3	*Pleurotus pulmonarius*	Radioactive cellulosic-based waste	Waste containing mushroom mycellium was solidified with portland cement and then this solidified waste act as first barrier against the release of radiocontaminants	Eskander et al. ([2012])
4	*Jelly* sp., *Schizophyllum commune* and *Polyporous* sp.	malachite green	99.75% (*Jelly* sp.), 97.5% (*Schizophyllum commune)*, 68.5% (*Polyporous* sp.2) dye was degraded in 10 days	Rajput et al. ([2011])
5	*Pleurotus pulmonarius*	crude oil	crude oil was degraded	Olusola and Anslem ([2010])
6	*Coriolus versicolor* MKACC 52492	PAH	Mushroom possesses ability to degrade Poly-R 478 which decides its suitability to degrade PAH. Lignin-modifying enzymes laccase, manganese-dependent peroxidase (MnP), and lignin peroxidase (LiP)was found to produce for degradation	Jang et al. ([2009])

Mushroom can produce extracellular peroxidases, ligninase (lignin peroxidase, manganese dependent peroxidase and laccase), cellulases, pectinases, xylanases and oxidases (Nyanhongo et al. [2007]). These are able to oxidize recalcitrant pollutants *in vitro*. These enzymes are typically induced by their substrates.

These enzymes have also been found to *degrade nonpolymeric, recalcitrant pollutants* such as nitrotoluenes (VanAcken et al. [1999]), PAHs (Hammel et al. [1991]; Johannes et al. [1996]), organic and synthetic dyes (Ollikka et al. [1993]; Heinfling et al. [1998]), and pentachlorophenol (Lin et al. [1990]) under in vitro conditions. Recently, it is reported that mushroom species are able to *degrade polymers* such as plastics (da Luz et al. [2013]).

The biodegradation mechanism is very complex. The reason is the influence of other biochemical systems and interactions of ligninolytic enzymes with cytochrome P_450_ monooxygenase system, hydroxyl radicals and the level of H_2_O_2_ which are produced by the mushroom.

### Biosorption

The second important process of removal of metals/pollutants from the environment by mushroom is - *biosorption*. Biosorption is considered as an alternative to the remediation of industrial effluents as well as the recovery of metals present in effluent. Biosorption is a process based on the sorption of metallic ions/pollutants/xenobiotics from effluent by live or dried biomass which often exhibits a marked tolerance towards metals and other adverse conditions (Gavrilescu [2004]). Biosorbents can be prepared from mushroom mycelium and spent mushroom compost.

The uptake of pollutants/xenobiotics by mushrooms involves a combination of two processes: (i) bioaccumulation i.e. active metabolism-dependent processes, which includes both transport into the cell and partitioning into intracellular components; and (ii) biosorption i.e. the binding of pollutants to the biomass without requiring metabolic energy. Several chemical processes may be involved in biosorption, including adsorption, ion exchange processes and covalent binding. According to Mar'in et al. ([1997]), the polar groups of proteins, amino acids, lipids and structural polysaccharides (chitin, chitosan, glucans) may be involved in the process of biosorption.

A lot of study has been done on the *biosorptive capacity of biomass* of mushroom and are shown in Table 3. It is reported that the biosorption capacity of dead biomass may be greater, similar to or less than that of living cells (Mar'in et al. [1997]). In the case of biosorption, dead biomass of mushroom offers certain advantages over living cells. Dead mushroom biomass can be obtained from industries as a waste of fermentation processes. Further, this is not sensitive to concentrations of toxicants and their toxicity effects and adverse operating conditions (pH, temperature, nutrient supply, initial metal ion concentration, and the concentration of cells etc.) unlike living mushroom biomass. The uptake of xenobiotic by living cells depends on fungal species and contact time. Biosorption techniques are now becoming very popular for the removal of pollutants. Biosorption is an effective method due to the high uptake capacity and very cost-effective source of the raw material.

**Table 3:** Removal of pollutants by biomass of mushroom using biosorption process

**S. no**	**Mushroom spp.**	**Pollutants**	**Remarks**	**References**
1	*Agaricus bisporus*, *Lactarius piperatus*	Cadmium (II) ions	Wild *L. piperatus* showed higher removal efficiency on Cd(II) ions compared to the cultivated *A. bisporus*	Nagy et al. ([2013])
2	*Fomes fasciatus*	Copper (II)	Mushroom is efficient in biosorption of Cu (II) ions and hot-alkali treatment increased their affinity for Cu (II) ions	Sutherland and Venkobachar ([2013])
3	*Pleurotus platypus, Agaricus bisporus, Calocybe indica*	Copper, Zinc, Iron, Cadmium, Lead, Nickle	Mushrooms are efficient biosorbent for the removal these ions from aqueous solution	Lamrood and Ralegankar ([2013])
4	*Flammulina velutipes*	Copper	Mushroom compost used as biosorbent for removing copper ions from aqueous solution	Luo et al. ([2013])
5	*Pleurotus tuber- regium*	Heavy metals	*Pleurotus tuber-regium* biosorb the pollutant heavy metals from the soil artificially contaminated with some heavy metals	Oyetayo *et al.* ([2012])
6	*Pleurotus ostreatus*	Cadmium	Mushroom possess biosorption capacity and mechanism of biosorption was observed	Tay *et al.* ([2011])
7	*Pleurotus sajor-caju*	heavy metal Zn	Mushrooms biosorb the heavy metals	Jibran and Milsee Mol ([2011])

#### Bioconversion

Nowadays, the research on conversion of industrial or agro-industrial sludges into some other useful forms is going on. The most important *bioconversion* product is - mushroom. Any lignocellulosic waste, generated by industries, can be used for cultivation of mushroom which can be further use as a product. Mushroom species cultivated on industrial and agro-industrial wastes are given in Table 4. The choice of the substrate for the cultivation of mushroom is generally determined by the regional availability of the material.

**Table 4:** Bioconversion of waste by mushroom species

**S. no.**	**Mushroom spp.**	**Bioconversion of waste**	**Remarks**	**References**
1	*Pleurotus citrinopileatus*	Handmade paper and cardboard industrial waste	Successfully cultivated. Basidiocarps possessed good nutrient content and no genotoxicity	Kulshreshtha et al. [(2013)]
2	*Pleurotus ostreatus*	Extract from the sawdust	Biomass of mushroom was produced in submerged liquid culture were analyzed	Akinyele *et al.* ([2012])
3	*Volvariella volvacea*	Agro-industrial residues such as cassava, sugar beet pulp, wheat bran and apple pomase	Enzyme activities were measured during the fermentation of substrates	Akinyele *et al.* ([2011])
4	*Pleurotus florida*	Handmade paper and cardboard industrial waste	Successfully cultivated. Basidiocarps possessed normal morphology and no genotoxicity	Kulshreshtha *et al.* ([2010])
5	*Pleurotus*	Cotton waste, rice straw, cocoyam peels and sawdusts of *Mansonia altissima, Boscia angustifolia* and *Khaya ivorensis*	Successfully cultivated with good crude protein, crude fat and carbohydrate contents in sporophores.	Kuforiji and Fasidi ([2009])
6	*Pleurotus eous* and *Lentinus connotus*	Paddy straw, sorghum stalk, and banana pseudostem	Waste successfully bioconverted by mushroom with good biological efficiency	Rani *et al.* ([2008])
7	*Pleurotus tuber-regium*	Nigerian trees; *Terminalia superba, Mansonia altissima, Holoptelia grandis* and *Miliciaexcelsa*	Grow on trees	Jonathan *et al.* ([2008])
8	*Pleurotus tuber-regium*	Cotton waste, sawdust of *Khaya ivorensis* and rice straw	Sclerotia propagated on groundnut shells and cocoyam peels with lipase and phenoloxidase; cellulase, carboxymethyl cellulase enzymatic activities	Kuforiji and Fasidi ([2008])
9	*Lentinula edodes*	Eucalyptus waste	Successfully convert this waste and qualitative and quantitative changes were also measured	Brienzo et al. ([2007])
10	*Lentinula edodes*	Vineyard pruning (VP), barley straw (BS), and wheat straw	Bioconversion of VP waste with shortest primordium formation, highest biological efficiency, highest yield and shortest production cycle (6 days)	Gaitán- Hernández et al. ([2006])
11	*Lentinula tigrinus*	Wheat straw	Characterize the production of lignocellulosic enzymes and bioconvert the wheat straw	Lechner and Papinutti ([2006])
12	*V. volvacea*	Banana leaves (*Musa sapientum lina*)	Efficient bioconversion with good yield	Belewu and Belewu ([2005])

Mushroom cultivation has also been successfully done on various industrial wastes (Singhal et al. [2005]; Kulshreshtha et al. [2010]; Dulay et al. [2012] and Kulshreshtha et al. [2013b]). Applications of mushroom as mycoremediation tool in the bioconversion of these industrial wastes into protein rich mushroom carpophores (fruiting bodies of mushroom), on one hand provides mushroom and on the other hand helps in solving pollution problems, which their disposal may otherwise cause.

### Feasibility of the mycoremediation tool and processes

It is extremely important to carry out *feasibility study* before starting a remediation project in order to determine the best conditions for the process and toxicity in the fruiting bodies. The most important parameters to define a contaminated site are: biodegradability, contaminant distribution, chemical reactivity of the contaminants, soil type and properties, oxygen availability and occurrence of inhibitory substances (Martín et al. [2004]). The success of mycoremediation is governed by three important factors- availability of mushroom, accessibility of contaminants and a conductive environment. Therefore, the knowledge on the physiology and ecology of the biological species or consortia involved and the characteristics of the polluted sites are decisive factors to select an adequate mycoremediation protocol (Martín et al. [2004]).

Mycoremediation of waste from the environment by mushroom has many *advantages* but at the same time it is a challenge for the researchers and engineers. Mycoremediation of wastes can be done in *in situ* and *ex situ* conditions. When it is carried out on site, it eliminates the need to transport the toxic materials to treatment sites. It is an environmentally friendly approach and needs only a small space, low cost, less skilled persons and can be applied easily in the field. In contrast to above, there are some *disadvantages* in applying this mycoremediation tool. Mushrooms require time to adapt to the environment and cleanup wastes. Mushroom cultivated on industrial wastes may possess toxicity/genotoxicity. Genotoxicity of mushrooms is influenced by genotoxicants that are present in waste used for their cultivation. Therefore, it is necessary to assess toxicity/genotoxicity of mushrooms if used for bioremediation purpose.

Toxicity level in the fruiting bodies is based on two facts, i.e. biodegradation and biosorption. Mushroom possesses the suitable enzymatic machinery for biodegradation which lead to the degradation of pollutants from the substrate and convert it into less toxic products. This renders the fruiting bodies safe for consumption. Recently, many papers have published which reported that mushroom not only able to degrade pollutants but also able to *reduce the toxicity or mutagenicity* (Kulshreshtha et al. [2013b]; Choi et al. [2013]; Malachová et al. [2006]). Numerous studies stated that mutagenicity reduction by mushrooms is primarily species dependent. Kulshreshtha et al. ([2011]) and Kulshreshtha et al. ([2013b]) reported *Pleurotus florida* was not found to have genotoxicity, however, *Pleurotus citrinopileatus* have had genotoxicity in their fruiting bodies when both were cultivated on industrial wastes and the mixture of wheat straw and industrial wastes under the same cultivation conditions.

Toxicity reduction is also dependent on the substrate. Same fungi may have *different capability in degrading the different pollutants* (Choi et al. [2013]) due to the enzymes of mushrooms that are not only involved in degradation but also reducing the effects of toxic and genotoxic pollutants. Several researchers have proved the antimutagenic and antigenotoxic power of mushroom (Mendez-Espinoza et al. [2013]; Taira et al. [2005]; Mlinaric et al. [2004]; Filipic et al. [2002]; Menoli et al. [2001]) which may be used to reduce the genotoxicity of the pollutants. Therefore, it is proved that besides having degradation power mushrooms can reduce the genotoxicants and toxic pollutants due to having antimutagenic and antigenotoxic power. These types of species of mushroom can be used for edible purposes and as feed for animals. This concept provides a natural guide to future research which should be focused on the need of research to degrade the pollutants in such a way that their disposal will not create another problem and fruiting bodies can be consumed safely. In contrast to this, *absorption of pollutants by mushroom makes them unsuitable for consumption*. Many researchers have reported the very high amount of metal content and mutagenicity in the fruiting bodies of mushrooms growing on polluted substrate, naturally or artificially, which is due to the absorption process (Tables 3 and 5). Wild Further information is needed about the level of toxicity in these mushrooms, ignorance of which will cause the associated health related problems.

**Table 5:** Mutagenicity of naturally occurring and cultivated mushroom species detected by Ames test

**S. no.**	**Mushroom types**	**Mutagenicity test results**	**Reference**
1	Nine wild and two cultivated species of Spanish edible mushrooms	The mushrooms were mutagenic to TA100 and TA98 strains	Morales *et al.*, ([1990])
2	Wild and commercially grown mushrooms	Presence of microsomal enzymes (S-9) reduced the mutagenic effects of all the mushrooms, with the exception of *Agaricus abruptibulbus* and *Cantharellus cibarius.*	Gruter *et al.*, ([1991])
3	*Agaricus bisporus*	Direct-acting mutagenic response in various *Salmonella typhimurium* strains, TA104. Agaritine is not responsible for the mutagenicity of mushroom extracts	Papaparaskeva *et al.*, ([1991])
4	*Agaricus bisporus*	Agaritine was weakly mutagenic, in the absence of an activation system, in *Salmonella typhimurium* strain TA104.	Walton *et al.*, ([1997])
5	*Pleurotus florida* cultivated on handmade paper and cardboard industrial waste	Not mutagenic with either TA 98 or TA 100 strain	Kulshreshtha et al., ([2011])
6	*Pleurotus citrinopileatus* cultivated on handmade paper and cardboard industrial waste	Mushroom extract is mutagenic with TA 98 strain	Kulshreshtha et al., [(2013)]

*Biosorption* can become a *good tool* to remediate toxic metals threatening the environment (Lamrood and Ralegankar [2013]) but on the other hand, this process *generates non-consumable biomass* which gives rise to the new problem of disposing it. Usually researchers have been focused on the use of mushroom mycelium for biosorption and compare the abilities of biomass for sorption (Table 3). A very few publications reported the reason of varying power of biosorption to various types of mushroom (Kumhomkul and Panich-pat [2013]; Das [2005]). This fact may be a decisive factor for further use of mushroom species.

It is proved that mushrooms have different abilities of biosorption, bioremediation, biodegradation and toxicity reduction. In my opinion, researchers should try to first remediate the heavy metals by cultivating high metal absorbing species of mushroom. However, low absorbing edible species can be used to cultivate on waste so that absorption of the pollutants can be minimized. Researchers should also try to develop the method of using biomass repeatedly for the biosorption of pollutants which will also reduce the waste generation. The toxicity or genotoxicity of these mushroom species should be assessed and thereafter, non-toxic mushroom species can be used for consumption. However, in the case of remediation of pollutants preference should be given to those species which can degrade the pollutants. The safe species will be selected to remediate a particular type of waste and further use for consumption.

## Conclusion

Mushroom is a tremendous boon to the idea of using this for mycoremediation process as a real-world solution. The cultivation of edible mushroom on agricultural and industrial wastes may thus be a value added process capable of converting these discharges, which are otherwise considered to be wastes, into foods and feeds. Besides producing nutritious mushroom, it reduces genotoxicity and toxicity of mushroom species. Mycoremediation through mushroom cultivation will alleviate two of the world’s major problems i.e. waste accumulation and production of proteinaceous food simultaneously. Thus, there is a need for further research towards the exploitation of potential of mushroom as bioremediation tool and its safety aspects for consumption as product.

## Competing interests

The authors declare that they have no competing interests.
